# Intracytoplasmic Sperm Injection (ICSI) in Extreme Cases of Male Infertility

**DOI:** 10.1371/journal.pone.0113671

**Published:** 2014-12-01

**Authors:** Gianpiero D. Palermo, Queenie V. Neri, Peter N. Schlegel, Zev Rosenwaks

**Affiliations:** 1 The Ronald O. Perelman & Claudia Cohen Center for Reproductive Medicine, Weill Cornell Medical College, New York, New York, United States of America; 2 Department of Urology, Weill Cornell Medical College, New York, New York, United States of America; University Hospital of Münster, Germany

## Abstract

**Introduction:**

Severely compromised spermatogenesis typical of men with virtual azoospermia or non-obstructive azoospermia requires an extreme search for spermatozoa. Our goal was to evaluate the usefulness of a meticulous search carried out in ejaculated or surgically retrieved specimens in achieving pre- and post-implantation embryo development.

**Patients and Methods:**

In a retrospective cohort study carried out in an academic institution, intracytoplasmic sperm injection (ICSI) outcomes were reviewed as a function of length of microscopic sperm search in ejaculated and surgically retrieved specimens. Couples whose male partner presented with either virtual or non-obstructive azoospermia were treated by ICSI and categorized according to the time spent in identifying and retrieving enough spermatozoa to inject all the oocyte cohort. Semen parameter, fertilization, pregnancies, deliveries, and child welfare in relation to increasing search time were analyzed and compared.

**Result(s):**

The maternal and paternal ages were comparable in both ejaculated and testicular sperm extraction (TESE) groups along with the oocytes retrieved. The fertilization rates for both ejaculated and TESE progressively decreased with increasing time (*P*<0.0001). Clinical pregnancies in the ejaculated cohort remained satifactory. In the TESE cohort, there was a decrease in pregnancy rate with increasing time, from 44% to 23%. In a limited number of cases, offspring health was evaluated in both semen sources and appeared reassuring.

**Conclusion(s):**

An extensive and at time exhaustive sperm quest yields kinetically and morphologically impaired spermatozoa without apparent impact on embryo developmental competence. Retrieval of spermatozoa from the seminiferous tubules provided more consistent fertilization and pregnancy outcomes than those retrieved from the ejaculate. A trend indicated that pregnancy rate decreased as search time increased in the TESE group. The utilization of the scarce and unselected spermatozoa did not obviously impair embryo development or cause post-implantation errors.

## Introduction

The introduction of intracytoplasmic sperm injection (ICSI) [1] has been responsible as now for over two million babies worldwide and has been instrumental in helping men with suboptimal gametes to achieve their reproductive dream [2], [3]. ICSI has supplanted all prior assisted fertilization techniques because it has the ability to successfully bypass cases with anti-sperm antibodies, to deal with sperm acrosome dysfunction, and to override sperm kinetic defects [4]. ICSI is not impacted by of the dys-maturity of the male gamete such as those generated from the epididymis and the testicle often characterized by an incomplete flagellum and peculiar cell membrane [5], [6]. These successes achieved across the arrays of dysfunctional spermatozoa has allowed to push the boundaries of the application of the sperm injection technique towards the most extreme aspect of male infertility even when only few spermatozoa can be identified as often encountered in cryptozoospermia, virtual azoospermia or when surgical specimens are used in absolute azoospermia [7]. The ability to obtain pregnancies with these scarce spermatozoa has introduced another variable that is the injection of the non-ideal and unselected spermatozoon. This, for some gamete purists, raises concerns due to genetic and epigenetic risks induced by the utilization of such unorthodox gametes [8], [9]. In fact, while for standard ICSI a popular trend has surfaced toward the selection of the most adequate spermatozoon screened for presence of head vacuoles, motile sperm organelle morphology examination or the expression of hyaluronan antigen as a sign of maturity of the male gamete [10], [11]. These screening efforts are aimed at identifying the euploid spermatozoon with intact chromatin having the best chances of contributing to normal embryo development [12]. While these methods, although unproven, are laudable and feasible only when adequate spermatozoa are available to select from. In virtual azoospermia cases, the pressing need is in the identification and retrieval of individual sperm cells. Thus an extended sperm search, in function of the time spent, shifts the paradigm toward overlooking the morphological selection and focusing on the actual presence of a sperm cell, possibly motile, to gauge viability status.

Counseling couples whose male partner has scarce spermatozoa in the ejaculate raises another question on whether semen specimens should be collected from a more proximal site of the male genital tract such as the seminiferous tubule [13]–[15]. This is to control for the presence of unfavorable factors such as the exposure to oxidative stress, presence of white blood cells, and decaying germ cells present in the ejaculate that may contribute to sperm DNA damage while impairing viability and motility [16]–[18]. While surgical sampling approach may seem justifiable to men with azoospermia it is challenging to propose it to men suffering from cryptozoospermia [19]. Testicular sampling for its invasive nature entails anesthesia risks, surgical complications, and tissue scarring therefore requiring a thorough and well-expressed patient consent [20], [21].

Here we evaluate the attainments in the hardship of endeavoring an extreme quest toward the elusive spermatozoon and what it entails. We appraise couple’s endurance toward learning about their diverse facets of male infertility. We described the implications and outcome of strenuous search to identify spermatozoa in the ejaculate or surgical specimens. Finally, we compare the clinical outcomes pre- and post-implantation of extreme ICSI according to the origin of the male gamete.

The utilization of such scarce and unselected spermatozoa often have a lower ability to fertilize but still yield rewarding pregnancy rates and reassuring offspring health.

## Patients and Methods

### Ethics

The retrospective cohort analysis on embryological and clinical outcome after ICSI insemination was conducted in accordance with the research protocol approved by the Committee of Human Rights Research Weill Cornell Medical College (WCMC) (IRB 1307014154) that did not require written/oral consent.

The ART children follow-up study was performed in accordance with the research protocol approved by the Committee of Human Rights Research WCMC (IRB 1303013730) where the parents agreed and signed a written consent to participate in a questionnaire survey and children who were 5 years of age that agreed to a venipuncture and in-house pediatric evaluation signed an “Assent for Minors”.

Patients undergoing ICSI agreed with the proceedings elucidated in the Clinical Informed Consent for Assisted Fertilization devised by the Ronald O. Perelman and Claudia Cohen Center for Reproductive Medicine (CRM), WCMC.

### Study description and patient characteristics

**Figure 1:**
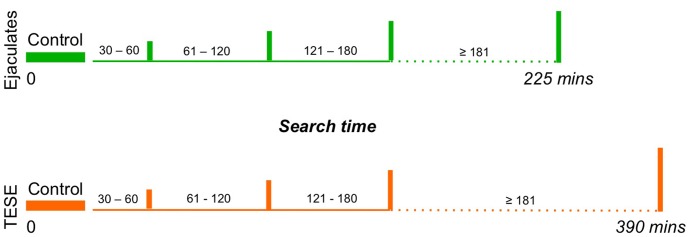
Time table depicting the allotted intervals for each study search group in relation to their respective control cohort for the ejaculated (green) and testicular biopsy (orange) sources.

A retrospective cohort analysis (IRB 1307014154) was conducted on ICSI cycles performed from September 1993 to December 2012 at CRM-WCMC. Couples whose male partner presented with either virtual or non-obstructive azoospermia were treated by ICSI and categorized according to the time spent in identifying and retrieving enough spermatozoa to inject all the oocyte cohort. ICSI cycles in which spermatozoa were retrieved from men with compromised spermatogenesis were included ranging from 500,000 spermatozoa all the way to azoospermia. Cycles were then evaluated in terms of the length of time necessary to retrieve sufficient spermatozoa for the ICSI procedure. The search was considered extended in cycles where the time to acquire all the needed spermatozoa to inseminate all the oocyte cohort took over 30 min all the way to several hours. The remaining cycles were regarded as controls (0–29 min) (Figure 1).

### Genetic screening

All couples with non-obstructive azoospermia (NOA) were offered genetic screening with molecular analysis for peripheral karyotype, Yq microdeletions, or aneuploidy assessment on sperm cells.

### Spermatozoa collection and processing

#### Ejaculates

Samples were produced after observing a standard 2–5 days of abstinence. Specimen that did not yield spermatozoa in the counting chamber were centrifuged and pellets placed in 8 µl microdrops under oil in ICSI dishes to be searched under an inverted microscope for presence of spermatozoa.

#### Testicular sampling

It is our Center’s policy that all men with NOA were requested to produce an ejaculate for semen analysis on the day of the scheduled testicular sperm extraction (TESE). If sufficient spermatozoa to be used for ICSI were identified in the ejaculate, then following discussion with the patient, surgery was cancelled. The microdissection approach was employed in order to enhance sperm retrieval while minimizing damage to the testicle as previously described [21]. The utilization of optical magnification allowed targeted identification of seminiferous tubules containing active foci of spermatogenesis and for selective removal of seminiferous tubules while sparing damage to vascularization [22].

#### Motility enhancement treatment

For spermatozoa with poor or absent kinetic characteristics, the sperm suspension was exposed to 0.35 mM pentoxifylline.

### Extensive sperm search

**Figure 2:**
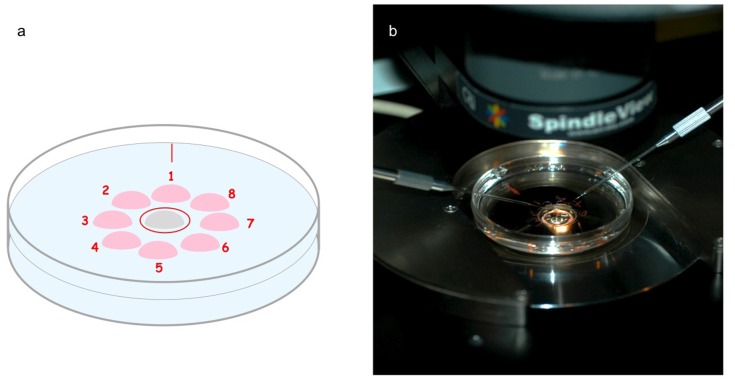
Schematic diagram of an ICSI dish containing a central drop of polyvinylpyrrolidone (PVP) and circled in red surrounded by 8-numbered drops (8 µl). A mark at 12 o’clock facilitates the start of the numbering counter-clock wise (**a**). An actual ICSI dish on a heated stage with microtools in position (**b**). For the control, spermatozoa were placed directly on the central drop and drops 1–8 used for oocyte allocation. In the study groups, the sperm samples were placed directly in descending order (e.g. drops 8, 7, 6) according the severity of the case. After retrieval of the spermatozoa from the peripheral drops, they would be moved and immobilized in the central PVP drop. Eventual residual drops free of sperm specimen would be used to allocate the oocytes or spermatozoa from the PVP would be transferred to a fresh dish with oocytes already in place.

Because of the extremely low concentrations of spermatozoa, the sperm search was carried out in ICSI dishes utilizing often all 8 drops available (Figure 2). Once spermatozoa were identified, they were transferred via an injection pipette to a central 7% polyvinylpyrrolidone (PVP) solution with human serum albumin (HSA) (90121, Irvine Scientific) containing drop. At the end of the search all spermatozoa were transferred to the PVP containing drop in a fresh ICSI dish for injection.

### Ovarian stimulation and oocyte preparation

Oocyte retrieval was performed after ovarian superovulation with gonadotropins and pituitary desensitization with GnRH-agonists or antagonists [23], [24]. The choice of a stimulation protocol was dependent on patient age, etiology of infertility, previous treatment history and physician preference. For all patients, one of several established stimulation protocols was utilized; lupron downregulation, microflare lupron or antagonist. Human chorionic gonadotropin (hCG) was administered (3,300–10,000 IU) when at least two follicles had reached or exceeded 16- to 17-mm diameter as observed by ultrasound. Dosage of hCG was tailored according to estradiol (E_2_) level and body mass index. Oocyte retrieval was performed approximately 35 to 36 hours after hCG administration via transvaginal needle guided aspiration. These oocytes were then exposed to 40 IU recombinant hyaluronidase (Cumulase, Halozyme Therapeutics, Inc. San Diego, CA) to remove cumulus-corona cells in a previously defined manner [24].

### Microinjection procedure

Details of the microinjection procedure have been previously described, including selection, immobilization and permeabilization of the spermatozoa [24], [25]. At all times it was necessary to substitute an 8 µl drop in the injection dish with 3 µl of the final sperm suspension.

Immediately before injection, 1 µl of the sperm suspension was diluted with 4 µl of PVP in HTF-HEPES medium placed in the middle of the plastic Petri dish. It was necessary to use the viscous solution during the procedure in order to slow down the motion of the spermatozoa and prevent it from sticking to the wall of the injection pipette. To load the search dishes, approximately 3 µl of concentrated sperm suspension was transferred directly into the injection dish in drop #8 (Figure 2) where each oocyte is placed in the remaining drops of G-MOPS (Vitrolife) supplemented with 6% G-MM (Vitrolife) and covered with lightweight oil (Sage Medical, Trumbull, CT, USA). Following immobilization, an individual spermatozoon was aspirated at the 3 o’clock edge of the PVP drop. For low concentration, a spermatozoon was retrieved by the injection tool from drop #8 and moved to the viscous medium central drop in order to remove debris, gain better aspiration control, and to carry out the immobilization [25].

It needs to be emphasized that the regular selection of spermatozoa for ICSI is performed under 400× magnification using best quality Nikon optics (MRH68400 CFI S Plan Fluor ELWD NAMC 40XC), this can be further enhanced, if opted, by a 1.5× with a built in magnifying lens and when needed higher “empty magnification” can be obtained by enlarging the image with the video imaging system (Nikon DS-Fi2; DS-L3). The spermatozoon is selected taking into consideration its head morphology as for presence for irregularities and imperfections, midpiece and flagellar shape. Dynamic characteristics included swimming patterns and progression as well as signs of membrane changes such as stickiness of the sperm head to the bottom of the dish or pipette.

For extreme searches, the spermatozoa identified in the search dishes as previously mentioned were transferred into the PVP containing drop of a new ICSI dish loaded with oocytes. For the cases requiring searches longer than 30 min and in those requiring hours the criteria for sperm selection were restricted to identification of the actual cell, preferentially displaying kinetic patterns.

Oocytes were examined 12–17 hours after the injection procedure to assess for normal fertilization, defined by the presence of two distinct pronuclei (PN) and two clear polar bodies. Evaluation for embryonic cleavage was performed every 24 hours [26]. Morphologically good quality embryos were transferred into the uterine cavity on the third or fifth day after the microinjection procedure [27].

### Pregnancy assessment and therapeutic implantation support

Starting on the day of oocyte retrieval, methylprednisolone (16 mg/day) and tetracycline (250 mg every 6 hours) were administered for 4 days to all patients. Progesterone administration (25–50 mg I.M./day) was started on day 3 after hCG administration and was continued until the establishment of pregnancy. A serum βhCG assay was performed 14 days after the ovum pick-up. A biochemical pregnancy was defined as a positive βhCG level that decreased prior to when an ultrasound could detect an implantation site. A clinical pregnancy was defined as the presence of a fetal heartbeat by ultrasound assessment during the 7th week of gestation.

### Children’s health and development

Malformations with surgical or functional impairment requiring a surgical intervention were considered major; while all others that did not impair daily function were considered minor [28]. A minor anomaly was distinguished from a normal variation if it occurred in <4% of the infants in the same racial group [29]. For the 20% of neonates born at our institution, a detailed physical examination was performed at birth. For children born elsewhere, reports were obtained from gynecologists, pediatricians, or both which included a detailed physical evaluation.

Consenting parents of all children aged 3 years (±6 months) completed the Ages & Stages Questionnaires (ASQ), a series of parent-completed developmental questionnaires spanning from birth to 5 years of age [30] (IRB 1303013730). Five key developmental areas – communication, gross motor, fine motor, problem solving, and personal-social – were evaluated in addition to an overall section addressing specific parental concerns. According to the child’s score, questionnaires were ranked as typical development or as needing further evaluation (i.e. ‘at risk’, clinical range) [31]. Cognitive abilities, socio-emotional development, and motor skill scores were standardized for the general population and corrected for ART children [32]. The incidence of 3–5 year old children that requires special education (considered ‘at risk’) nationwide is 11.4% (http://disabilitycompendium.org/compendium-statistics/special-education).

### Statistical analysis

Statistical comparison to evaluate all relevant hypotheses was carried out by χ^2^ analysis, two-tailed at 5% level of significance using the Statview software (SAS Institute, Cary, NC, USA). Where appropriate, Fisher’s exact tests were used to ensure no violation secondary to the small cell counts in χ^2^ procedures. Student’s *t*-test was used to compare means using the SPSS statistical software (SPSS Inc., Chicago, IL). Multivariable analyses for a number of outcomes were adjusted for maternal age, paternal age, peak E_2_ level, and number oocytes retrieved were also performed. The analyses were performed separately in the ejaculated and the TESE groups. Statistical differences were recorded in text and tables only when reached.

## Results

### Treatment allocation and consenting

**Figure 3:**
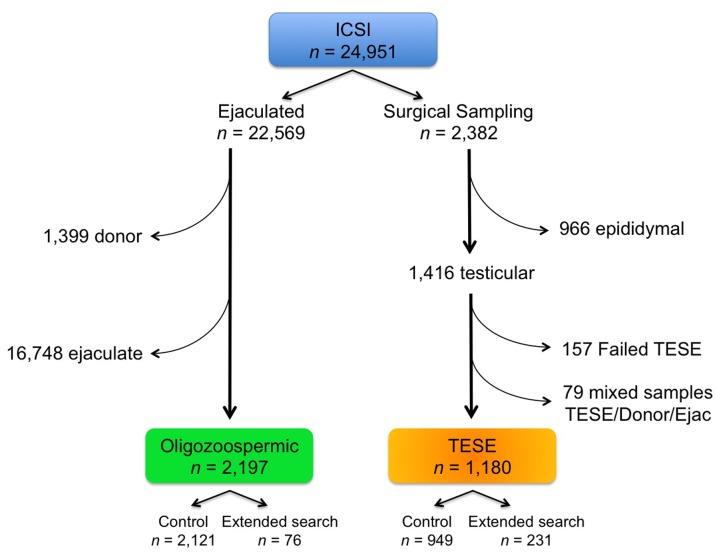
Flow chart explaining the allocation of ICSI cycles. In the ejaculate section, the number of donor specimens, standard ejaculates, and the severe oligozoospermic group included in the study are listed. For the surgical sampling portion, we showed the cycles where epididymal and testicular spermatozoa were used involving the group included in the present study.

**Figure 4:**
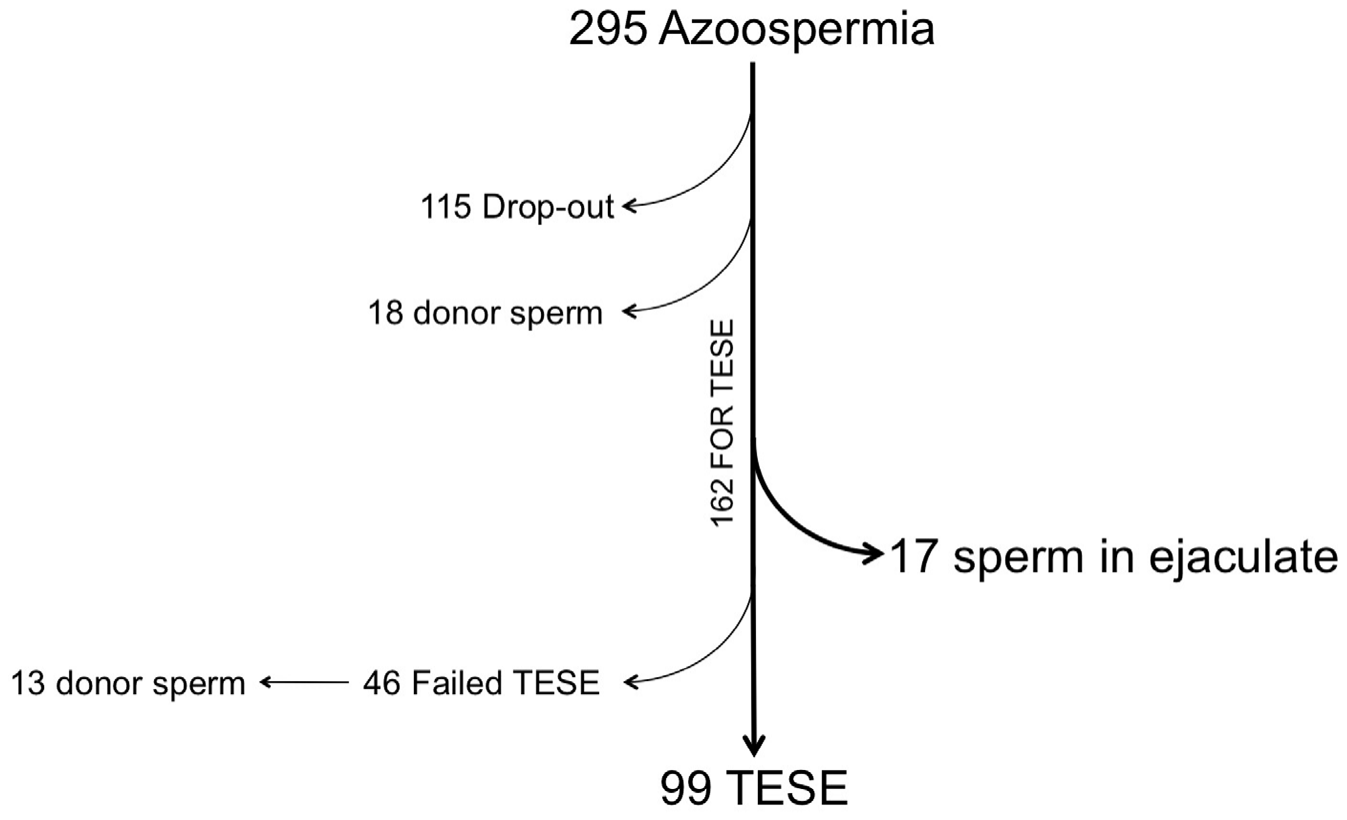
The diagram depicts the patients’ choice once their diagnosis of azoospermia was disclosed. It includes patient drop-out, those that opted for donor spermatozoa, and the patients that went through with TESE once their pre-operative ejaculates were screened and evaluated.

To summarize the treatment allocation of the patients included in this study, a flow chart has been provided (Figure 3). We thought that it would be interesting to explore patient response at consultation once the severe male factor status is revealed to the couple. We identified a sample of 295 patients that were screened in the andrology laboratory by semen analysis or extended sperm search. Since these men were recognized as pseudo-azoospermic, 133 (45%) of them decided to drop out and desist from their wish to have a child through ICSI. Meanwhile 18 of these couples proceeded with their ART enrollment but decided to use donor specimen for their 34 ART cycles ultimately achieving a clinical pregnancy rate of 52.9%. This left 162 (59%) couples whose male partners were counseled by their Reproductive Urologist and decided to proceed with testicular surgical sampling. As stated, the morning of the TESE attempt, it was our policy to examine the ejaculates of men undergoing surgical sampling and this approach allowed 17 (10.5%) men to avoid surgery because spermatozoa were identified in their ejaculates that, following ICSI, resulted in a clinical pregnancy rate of 44.0% (11/25). Of the final 145 men that underwent surgery unfortunately, microdissection of their seminiferous tubules failed to yield spermatozoa in 46 (28.4%) of them. On the day of the oocyte retrieval the inability to identify testicular spermatozoa induced 13 couples to use donor sperm in 39 cycles with 38.5% pregnancy rate. In 99 couples, testicular biopsy successfully yielded spermatozoa that were used for ICSI and resulted in 38.0% (60/158) clinical pregnancy (Figure 4).

### Genetic screening

Of the 1,536 extreme oligo- or azoospermic men screened in the Urology Department for TESE, 261 (17.0%) were found to have abnormal karyotypes with the large majority being Klinefelter (*n* = 170) followed by Yq microdeletions (50 AZFc, 7 AZFb, 3 AZFb+c, 3 gr/gr, and 1 small segment of Y chromosome), and the remaining being various combinations of autosomal abnormalities (inversions and translocations). All female partners had normal peripheral chromosomal constitution.

### Male gamete identification and retrieval

The total length of time utilized to search for a sufficient number of spermatozoa to inject all available oocytes ranged from 30 to 225 minutes (3.75 hours) for the ejaculated and all the way to 6.5 hours for the surgically retrieved spermatozoa (mean search of 116.2±42 mins) (Figure 1). In general, the searches utilized approximately three ICSI operators and sometimes up to eight additional embryologists (average number of personnel 4±2). Among all the cases and independently of the semen origin, 72.1% (220/305) of all cycles utilized a motility enhancer.

The couple characteristics and the treatment cycles for the ejaculated and the TESE samples are depicted in Table 1. For both ejaculated and testicular retrieval, the sperm search that took within 30 minutes were considered as control while the reminder of the cycles were allocated according to the increasing search time. As expected the cycle size decreased with the increasing time spent for spermatozoa searches and this was commonly observed in both sperm origin. Male age although higher than the female age did not noticeably vary among the groups. Importantly, the average maternal age, although unselected, was not higher than 35 years (except for the ejaculated cohort at ≥181 mins search time) and this allowed us to more reliably compare the study groups in relation to the male gamete characteristics while minimizing the eventual confounding female contribution.

**Table 1:** Patient demographics and gamete characteristics are grouped according to sperm source and length of sperm search.

	Ejaculated (mins)					TESE (mins)					
No. of	Control	30–60	61–120	121–180	≥181		Control	30–60	61–120	121–180	≥181
	1–29						1–29				
Couples	1,109	45	21	5	2		868	67	94	44	21
Male age (yrs)	34.9±5	37.7±7	37.1±9	43.3±12	40.4±7		38.5±8	37.6±10	36.2±8	35.9±8	35.2±6
[]×10^3^/ml	54,200	4,300	17	0.006	0.003		24.0	0.017	0.007	0.002	0.001
[Sperm seen] (range)	1−500	1–90	1–40	1−25	1−3		5–2800	2–900	1–100	1–25	1–7
Motile sperm (range)	1–82	0–19	0–4	0–1	0 −2		5–440	1–25	0–4	0–4	0–1
Cycles	2,121	48	21	5	2		949	68	98	44	21
Female age (yrs)	30.9±4	34.0±5	30.5±4	28.4±5	36.7±2		33.9±6	32.7±6	32.3±5	32.0±5	32.6±5
Total oocytes	26,184	580	303	75	25		11,284	904	1,430	712	379
Metaphase II	9.6±5	10.5±5	12.4±5	12.4±3	12.5±4		9.3±5	10.9±6	11.3±6	13.5±7	14.1±8

Note: Values are presented as mean ± SD; [ ] = concentration.

In crypto−/azoospermic men, the initial semen analysis is routinely carried out in a counting chamber that consistently failed to evidence presence of spermatozoa, therefore, the sperm parameter provided are derived following sample processing and selection. The average sperm concentration per thousand as well as the range of actual spermatozoa seen in cycles belonging to each time frame of the study groups were listed and compared to the controls for both sperm sources (Table 1). Similarly, we considered the actual motile spermatozoa identified just at the moment of injection. As expected these motile cells became progressively more scarce in the cases requiring a longer search time. As predicted by the homogeneous maternal age distribution, the average number of oocytes remained constant among the search time groups (Table 1).

**Figure 5:**
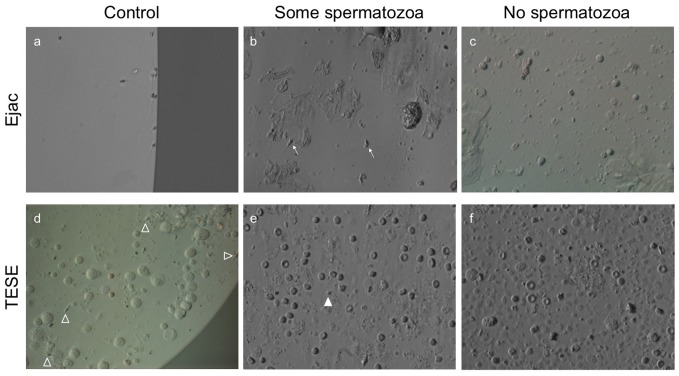
Specimen of a standard ejaculate evidenced spermatozoa swimming at the edge of the central PVP drop (a), specimen included in the study group where some spermatozoa (arrows) were seen (b) and where no spermatozoa were identified but presence of round cells, cellular debris, and epithelial cells were observed (c). Control testicular specimen was mechanically minced with spermatozoa (arrowheads) and immature germs cells present in a peripheral drop of medium (**d**). Scarce spermatozoon (solid arrowhead) with red blood cells and cellular debris in collagenase treated specimens placed in medium drops (**e**). Digested specimen with some interstitial cells, red bloods, cellular debris but without spermatozoa (**f**).

Depending on the sperm origin in question, the number of spermatozoa identified varied according to the time dedicated to the search. In most ejaculated specimens, particularly the control group, spermatozoa glided to the edge of the drop and were easily assessed for adequate morphology (Figure 5a). However, there were cases with severe cryptozoospermia where after ultracentrifugation spermatozoa were hidden by other cells (Figure 5b) and at times, no spermatozoa were identified among other cells and debris (Figure 5c). In testicular specimens, the same situation was observed in the controls where spermatozoa with acceptable phenotype were seen and picked up for injection (Figure 5d) while others not as easily identifiable (Figure 5e) and seldom, none at all were found except for cellular debris, red bloods cells, and interstitial cells (Figure 5f). Of the total TESE cycles (*n* = 1,416) in 157 instances no spermatozoa were identified even after an exhaustive sperm quest and the oocyte retrieved were injected with donor spermatozoa, cryopreserved, or disposed as per patient request.

### Embryological evaluation

Fertilization characteristics and rates are described in Table 2. Most relevant was the difference in fertilization between the two semen sources in the control groups (*P* = 0.0001) possibly due to the different gamete maturation and membrane characteristics. In both sample origins, there was a progressive decrease in normal fertilization with advancing search time (*P* = 0.0001). For the testicular specimens, the more scarce spermatozoa yielded reduced diagynic [33] rates (*P*<0.01) while providing a correspondent increase in oocyte activation void of male gamete participation (*P*<0.01), and an increasing proportion of oocytes that failed to fertilize (*P* = 0.0001) as the search time became lengthy.

**Table 2:** For ejaculated and testicular specimen sources, fertilization patterns, embryo quality, together with implantation are provided for the control and each sperm search time.

	Ejaculated					TESE				
No. of	Control	30–60	61–120	121–180	≥181	Control	30–60	61–120	121–180	≥181
	1–29					1–29				
MII injected	20,324	502	246	62	18	8,755	745	1,111	554	251
2PN (%)	15,359	282	129	21	11	5,141	372	505	154	67
	(75.6)^a^	(56.2)^a^	(52.5)^a^	(33.9)^a^	(61.1)^a^	(58.7)^b^	(49.9)^b^	(45.5)^b^	(27.8)^b^	(26.7)^b^
3PN (%)	504 (2.5)	11 (2.2)	6 (2.4)	0	0	268 (3.1)^c^	16 (2.1)^c^	21 (1.9)^c^	7 (1.3)^c^	1 (0.4)^c^
1PN (%)	473 (2.3)	11 (2.2)	11 (4.5)	0	0	453 (5.2)^d^	36 (4.8)^d^	56 (5.0)^d^	49 (8.8)^d^	14 (5.6)^d^
Transfers	1,917	44	19	4	2	865	59	76	34	15
Embryos tx (M)	4,129 (2.2)	109 (2.5)	33 (1.7)	8 (2.0)	4 (2.0)	2,323 (2.7)	141 (2.4)	158 (2.1)	70 (2.1)	32 (2.1)
D3 blastomeres	7.2±3	6.8±2	7.4±1	6.8±2	7.3±2	6.9±1.2	7.0±1.1	7.2±1.2	6.4±0.9	7.1±0.8
Fragmentation (%)	8.3±5.7	8.1±8.2	6.5±4.9	7.8±7.5	12.2±14	7.7±9.0	8.0±8.4	7.5±9.0	7.6±10.5	8.5±9.5
Implantation (%)	1202 (29.1)^e^	21 (36.8)^e^	16 (48.5)^e^	0^e^	1 (25.0)^e^	562 (24.1)	39 (27.7)	45 (28.5)	14 (20.0)	6 (18.8)

Note: Values are presented as mean ± SD; tx = transferred; M = mean.

a χ^2^ analysis of normal fertilization using ejaculated specimens according to increasing search time, *P*<0.0001.

b χ^2^ analysis of normal fertilization using testicular specimens according to increasing search time, *P*<0.0001.

c χ^2^ analysis of testicular cycles and decreasing 3PN formation with increasing search time, *P*<0.0001.

d χ^2^ analysis of testicular cycles and increasing 1PN formation with increasing search time, *P*<0.0001.

e χ^2^ analysis, implantation ability of zygotes generated from ejaculated samples, *P* = 0.028.

A multivariable analysis and adjustment for covariates confirmed our initial findings.

We then decided to compare the fertilization rates achieved at different search time slots for the two spermatozoal source, whether collected from the ejaculate or the seminiferous tubules. The fertilizing performance of the ejaculated spermatozoa seemed to out perform those retrieved from the seminiferous tubules throughout the search groups (Table 2).

Interestingly, embryo quality remained substantially unaffected throughout the different search times and this was common for both the spermatozoal origin (Table 2).

### Pregnancy characteristics

**Figure 6:**
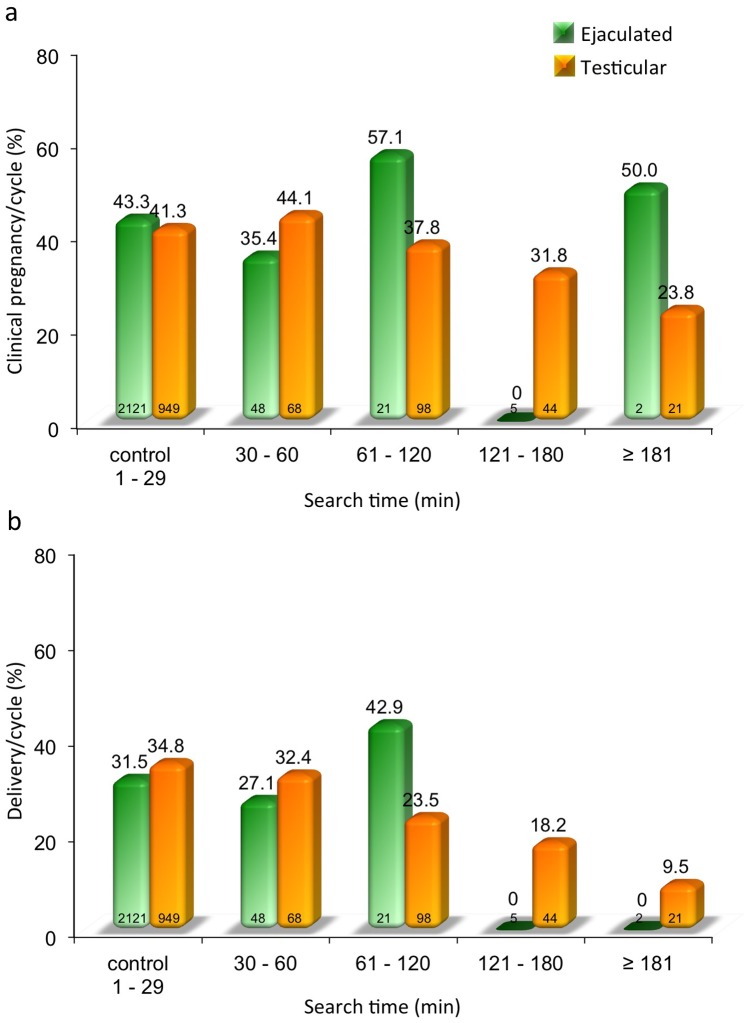
A comparison of clinical pregnancy outcome for the ejaculated (green) and testicular spermatozoa (orange) for each specific search group in relation to their respective controls is portrayed. (**a**). The proportion of deliveries with ICSI utilizing ejaculated and testicular spermatozoa are presented in relation to the sperm search time and controls (**b**).

To better evaluate the ability of these suboptimal and rare spermatozoa to contribute to embryo development, we grouped the clinical pregnancies according to the increasing search times. Interestingly, even with some fluctuation related to the search length for ejaculated specimens, clinical pregnancies remained unaffected between the control and study groups (Figure 6a). In addition, within the first 2 hours of search in this sperm source, there was no negative impact on the embryo implantation just as long as viable spermatozoa were identified and injected. In the testicular sampling, a decrease in clinical pregnancies appeared earlier following the one hour search (Figure 6a) and surprisingly, even for the most lengthy searches that extended over 3 hours, pregnancies, although somewhat lower, were not significantly impaired. When we looked at the pregnancies that proceeded to term, the delivery rate was consistent for the increasing search time when spermatozoa from the ejaculate were used (Figure 6b). The delivery rate of pregnancies generated from testicular spermatozoa showed a slight impairment proportional to the lengthening search, without however reaching mathematical significance (Figure 6b).

For both sperm origins the implantation rate followed a similar profile as the clinical pregnancies characterized by a slight decrease in the ability of the embryo to implant as the search time became longer (Table 2). However, pregnancy losses including biochemical, blighted ova and miscarriage were similar between the extended and control groups.

### ICSI children wellbeing

Among our offspring a neonatal malformation rate of 2.3% was reported without any correlation with the sperm source or the search time. The overall abnormality rate with the ejaculated was 2.6% (31/1182; 20 for minor and 11 for major malformations) and for TESE was 2.0% (22/1094; 14 minor and 8 major malformations).

In a delimited group of offspring that agreed to participate to a follow-up study aimed at assessing the psychological and physical development of children at 3 years of age generated at our center, we compared ICSI offspring to those generated through *in vitro* insemination. For both insemination procedures the incidence of children at risk was 10.4% (26/251) for IVF versus 11.4% (55/481) for ICSI with ejaculated) that is in line with the general population. Interestingly, the offspring generated with testicular spermatozoa, only 2.8% (2/71) of them were at risk of developmental abnormalities and required further evaluation.

## Discussion

In the last few years the treatment of severe male factor has evolved so dramatically that the limitation is not in the clinical indication or in some sort of conventional threshold but is purely related to the ability to retrieve spermatozoa in the specimen [13], [34]. The success of this approach is then related to the personnel available to tackle the task, in the ability to correctly dilute the specimen in the drops under oil, and in the determination in going the extra mile to identify the sought after spermatozoon. The unpredictable difficulty in identifying and retrieving spermatozoa with a varying number of embryologists available to simultaneously search at a given time may represent a bias in this analysis. At times, these quests are so extreme that not all spermatozoa needed to inject the harvested oocytes are identified even after several hours of search. This becomes particularly onerous on ICSI operators, andrologists, and embryologists involved and can be perceived by the individuals involved as the bleak aspect of their profession. This is felt even more when many such cases are concentrated in a laboratory such as ours operating in a referral center highly specialized for these severe male factor procedures and tightly intertwined with the Reproductive Urology service.

The success of this work should also be considered in terms of technician and overall operational costs that eventually should be explained to the patients or the third party responsible for insurance coverage [35]. The intent of this analysis is to provide feedback on the ability to identify limitations and circumstances that would guide the halting of a sperm search. Because there is no way to guarantee the detection of spermatozoa on the day of ovum pick up, the clinical decision regarding the source of the gamete to be used in a particular ICSI cycle is based on the presence or absence of the actual sperm cells in the ejaculate. Thus, the shift is represented by the actual identification of spermatozoa and less so by its morphological characteristics [11]. Moreover, our analysis did not seem to help in identifying a search time beyond which the sperm quest would be futile.

In extreme ICSI the sperm identification needs to be carried out in the actual sample at the time of oocyte injection because of the inherent inability of repeated semen analyses or diagnostic biopsies to provide a reliable picture on the availability of sperm in the specimen [36]–[38]. Furthermore the cryopreservation of these poor specimens is not helpful unless carried out in beads, artificial vectors, in microdrops, or chips [39]–[44]. Finally, the ability of ICSI to utilize virtually any spermatozoon eventually identified render the abovementioned efforts almost irrelevant [4].

When treating NOA patients, that often spill some spermatozoa in the ejaculate, we are often faced with the dilemma of taking the challenge of using the extremely few cells from the ejaculate versus recommending the testicular biopsy approach. Obviously, clinicians and patients are reluctant to immediately use testicular spermatozoa because it mandates an invasive surgical procedure with irreversible damage to the tissue. To this end, it is helpful to tailor patient counseling by providing more information regarding the performance of spermatozoa, from different provenance, in terms of clinical outcome [13], [14]. To elucidate patient response to this information, we have provided a flow chart (Figure 4) from where it was possible to gauge the decisional behavior of the couple once informed about the actual cause of their infertility. This study carried out at our clinic depicts couples’ attitude and approximately half decided to opt-out from treatment therefore renouncing to have their own biological child as demonstrated by the 13.5% that underwent donor gamete use. For the couples that decided to go ahead with the uncertain chances of retrieving spermatozoa, 28.4% of them failed to have sperm retrieved by TESE and their acceptance of using donor gametes rose to 28.3% [14]. This indicated a stronger commitment to having a child, regardless of their own genetic contribution in the group that underwent testicular biopsy and failed to identify spermatozoa. The ability of ICSI to successfully utilize any spermatozoa independently of their origin, motility, and morphological structure allow to minimize the utilization of donor gametes limiting it only for these unfortunate instances [14].

From this analysis, it appears that men that plan to undergo testicular biopsy and have spermatozoa identified in the ejaculate the morning of the procedure can hold off surgery while men that proceed to TESE may still fail to yield spermatozoa. The lesson to be shared with the patient is that once spermatozoa for ICSI are identified, whether in the semen or the seminiferous tubules, they have a similar chance to generate an offspring [34].

In the paper by Ben-Ami and colleagues [45], clearly discriminates the terminology of crypto-zoospermia versus virtual azoospermia, where the latter is defined as the category of men that inconsistently present spermatozoa in their ejaculates. In our view, these two terms are verisimilar in describing the same condition. In fact, fertile men present with a large fluctuation in semen values [46], [47], and this should be particularly evident in men that invariably display a handful of spermatozoa in their semen.

From the current and previous analyses [13], [19], we have not been able address who should be included in extreme ICSI alluding to a particular male infertility profile. In fact, the last word is delegated to the presence of an individual spermatozoon within the specimen. The next question is how long should the search last and this is currently answered by the number of spermatozoa identified for injection of the oocyte cohort. However, in instances where after one hour of search by 4 highly trained ICSI operators or to sample exhaustion and not a single spermatozoon is identified, the sperm search should be stopped.

Our analysis identifies differences in terms of fertilization that progressively decreases over time (Table 2). The fertilization was slightly superior for the ejaculate specimens and may be explained by the fact that a spermatozoon that has completed his journey in the male genital tract undergoing all the maturational membrane changes yield a fully formed and more competent sperm cell [5]. The fertilization obtained here with the injection of the scarce spermatozoa retrieved was higher in the ejaculated group than the surgical one contrary to other studies [45]. The satisfactory performance in terms of clinical pregnancy and delivery rates for ejaculated and testicular spermatozoa chosen at different search times is also somewhat surprising (Figure 6a,b). Nevertheless, we recognize that in the longer search-time groups the limited number of cycles render the clinical pregnancy and delivery proportions inconsistent specifically in the ejaculated cohort. This implies that the conventional criteria generally used to select a spermatozoon for ICSI are overlooked when we are dealing with a handful of spermatozoa following several hours of microscopic observation. These findings differ somewhat from our prior work [34] where a testicular cycle was compared to the ejaculated closest in time within the same individuals and the fertilization was comparable between the two different sources. This may be explained by the fact that in the prior paired series the spermatozoa performance belonged to the same individual while in this work includes a comparison between men that have a diverse spermatogenetic dysfunction in their germinal epithelium.

This study has important implications regarding the attempt of selecting the healthiest spermatozoon to be injected. In extreme ICSI, couples are extensively counseled about their condition and if eventual sperm cells are retrieved the gamete will be selected according to basic characteristics such as the presence of a head and one flagellum, preferentially motile. In the eventuality that no motile cells are identified, those will be used. Because of the impossibility to morphological select these spermatozoa it would be reasonable to expect that the clinical outcome would be extremely poor, but to our surprise instead the participation to embryo development of these gametes is comparable to spermatozoa carefully selected. This is in line with our previous experimental work focused at addressing the validity of high magnification to identify spermatozoa for ICSI [48]. In a multicenter collaborative study, we were unable to confirm the benefit of high magnification selection method not only in terms of improving pregnancy chances but also in the ineffectiveness in enhancing the yield of spermatozoa with correct chromosomal content or intact chromatin [48], [49]. Furthermore, this was also observed with the hyaluronan selection [50].

In previous observations [4], [51], we have clearly and repeatedly demonstrated the correlation of chromatin integrity and spermatozoa motility. This is supported by the clear relationship of a low DFI and pregnancy outcome when natural intercourse and IUI are used [49], [52], [53]. Instead this correlation become inconsistent when *in vitro* insemination is used and almost non-existent when ICSI is chosen [4], [49], [54]. In fact the latter two procedures require the motile spermatozoa portion of a specimen to reach the oocyte, spontaneously or by direct injection [49], [55]. Indeed even with extreme ICSI, we preferentially used spermatozoa that display motility characteristics. In fact, when motility is present even if ranging from progressive, to in place, or simply twitching – this display is proof of cell viability. This approach would explain the retained embryo developmental competence of the unselected scarce spermatozoa used in this study. Once again in support of the fact that as it is true for oocytes and embryos, the spermatozoa phenotype is not absolutely correlated to its genotype or epigenomic conditions.
